# High Spatial Resolution Multi-Organ Finite Element Modeling of Ventricular-Arterial Coupling

**DOI:** 10.3389/fphys.2018.00119

**Published:** 2018-03-02

**Authors:** Sheikh Mohammad Shavik, Zhenxiang Jiang, Seungik Baek, Lik Chuan Lee

**Affiliations:** Department of Mechanical Engineering, Michigan State University, East Lansing, MI, United States

**Keywords:** left ventricle, finite element modeling, ventricular-arterial coupling, arterial mechanics, cardiac mechanics, systemic circulation

## Abstract

While it has long been recognized that bi-directional interaction between the heart and the vasculature plays a critical role in the proper functioning of the cardiovascular system, a comprehensive study of this interaction has largely been hampered by a lack of modeling framework capable of simultaneously accommodating high-resolution models of the heart and vasculature. Here, we address this issue and present a computational modeling framework that couples finite element (FE) models of the left ventricle (LV) and aorta to elucidate ventricular—arterial coupling in the systemic circulation. We show in a baseline simulation that the framework predictions of (1) LV pressure—volume loop, (2) aorta pressure—diameter relationship, (3) pressure—waveforms of the aorta, LV, and left atrium (LA) over the cardiac cycle are consistent with the physiological measurements found in healthy human. To develop insights of ventricular-arterial interactions, the framework was then used to simulate how alterations in the geometrical or, material parameter(s) of the aorta affect the LV and vice versa. We show that changing the geometry and microstructure of the aorta model in the framework led to changes in the functional behaviors of both LV and aorta that are consistent with experimental observations. On the other hand, changing contractility and passive stiffness of the LV model in the framework also produced changes in both the LV and aorta functional behaviors that are consistent with physiology principles.

## Introduction

The heart and vasculature are key components of the cardiovascular system that operate in tandem to deliver oxygen and nutrients to the human body. Physiological adaptation, deterioration, and/or malfunctioning of one component often affects the operation of the other. Indeed, optimal ventricular-arterial interaction (or coupling) is critical to the normal functioning of the cardiovascular system. Any deviations from optimal ventricular-arterial interaction in the cardiovascular system (as indexed by the ratio between arterial stiffness and ventricular elastance) are usually associated with heart diseases (Borlaug and Kass, 2011). In the pulmonary circulatory system, interactions between the right ventricle and the pulmonary vasculature are key determinants of the clinical course of pulmonary hypertension (Naeije and Manes, 2014), specifically, in the transition from compensated to decompensated remodeling. Similarly, in the systemic circulatory system, heart failure with preserved ejection (HFpEF) has been associated with a progressively impaired ventricular-arterial interaction between the left ventricle (LV) and the systemic arteries (Kawaguchi et al., 2003; Borlaug and Kass, 2011). Ventricular-arterial interaction is also reflected at the microstructural level. In particular, remodeling of the vasculature found in these diseases (e.g., smooth muscle hypertrophy/proliferation and deposition of the collagen) (Shimoda and Laurie, 2013; Giamouzis et al., 2016) are often accompanied by similar remodeling in the heart (e.g., myocyte hypertrophy and cardiac fibrosis) (Rain et al., 2013; Hill et al., 2014; Su et al., 2014).

Computational modeling is particularly useful for understanding ventricular-arterial interaction, especially as there are potentially many parameters that can affect this interaction bi-directionally. While ventricular-arterial interactions may be described using electrical analog (or lumped parameter) models of the cardiovascular system (Smith et al., 2004; Arts et al., 2005), the heart and vasculature in such models are represented using highly idealized electrical circuit elements such as resistor, capacitor, and voltage generator. It is difficult, if not impossible, to separate or distinguish between geometrical, material, and microstructural changes from the parameters of these electrical elements. Previous finite element (FE) modeling efforts of the cardiovascular system, however, have focused on either the heart or the vasculature. Specifically, FE models of the heart were developed either in isolation (Wenk et al., 2011; Lee et al., 2013; Gao et al., 2014; Genet et al., 2014), or coupled to an electrical analog of the circulatory system in open (Usyk et al., 2002; Trayanova et al., 2011; Wall et al., 2012; Lee et al., 2016; Xi et al., 2016) or closed loop fashions (Kerckhoffs et al., 2007; Shavik et al., 2017). In an open-loop circulatory modeling framework, the FE ventricular model is generally coupled to a Windkessel model via outlet boundary conditions to simulate the ejection of blood, while the filling and isovolumic phases are, respectively, simulated by increasing and constraining the ventricular cavity volume. Parameters in the modeling framework are then adjusted so that the four distinct cardiac phases form a closed pressure-volume loop. On the other hand, coupling the FE ventricular model to a closed loop circulatory modeling framework is (arguably) more physical since the total blood volume is naturally conserved in the cardiovascular system. Simulation of multiple cardiac cycles is required, however, to obtain a steady state solution. Conversely, FE models of the vasculature were developed either in isolation (Hsu and Bazilevs, 2011; Zeinali-Davarani et al., 2011) or coupled to simplified representation of the heart based on a time-varying elastance function (Kim et al., 2009; Lau and Figueroa, 2015). Although able to describe the heart or vasculature in greater details, these FE modeling frameworks cannot be used to simulate detailed bidirectional ventricular-arterial interactions e.g., how changes in the vasculature mechanical properties affect the deformation and function of the heart and vice versa.

To overcome these limitations, we describe here a novel computational framework that is capable of coupling high spatial resolution FE models of both the vasculature and the heart to describe bidirectional ventricular-arterial coupling in the systemic circulation. Using realistic geometries and microstructure of the LV and aorta, we show that the framework is able to reproduce features that are consistent with measurements made in both compartments. We also performed a parameter study to show how mechanical and geometrical changes in the aorta affect the heart function and vice versa.

## Methods

### Closed-loop systemic circulatory model

**Figure 1:**
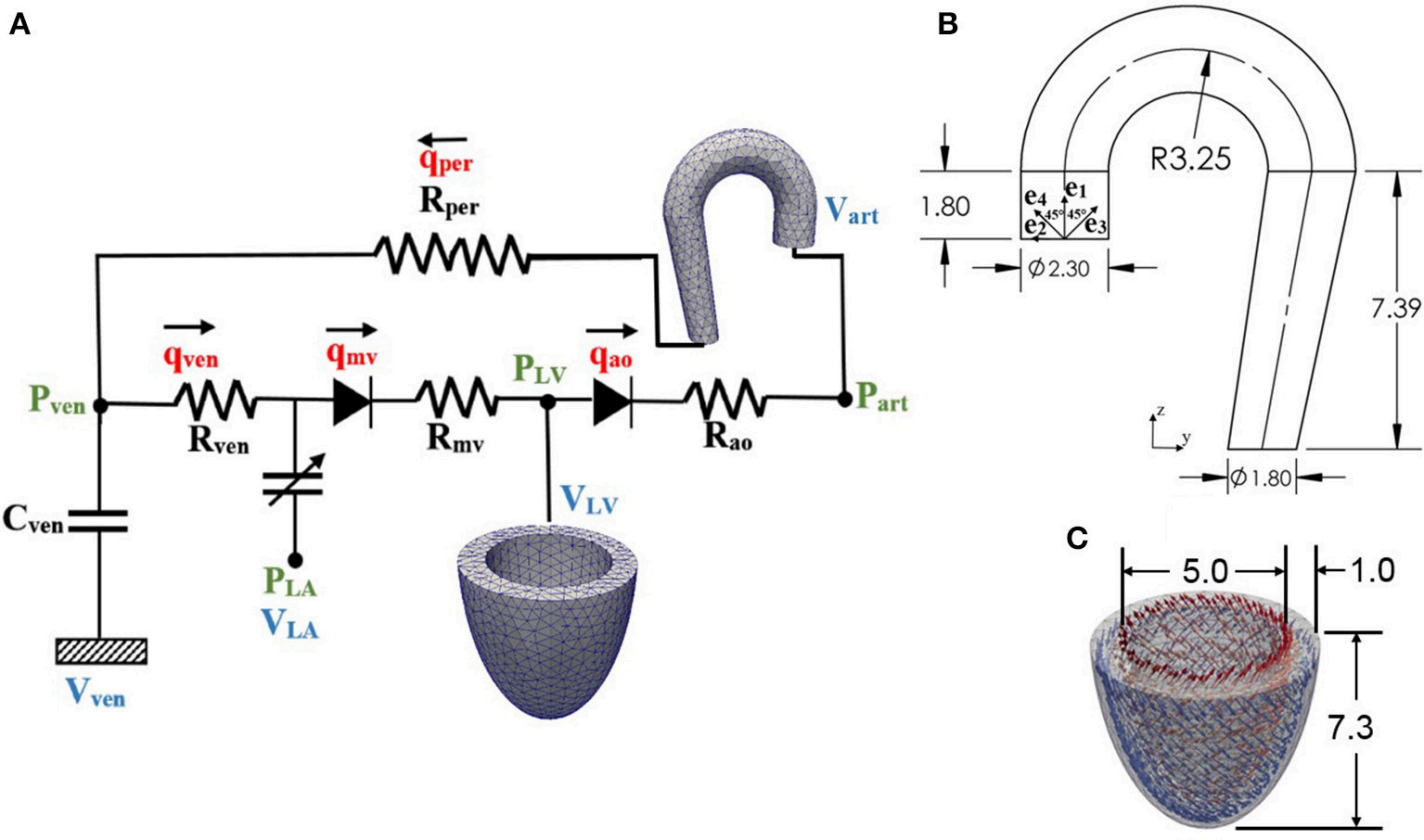
**(A)** Schematic diagram of the ventricular-arterial modeling framework; the LV and aorta were modeled using FE models, rest of the systemic circulation compartments were modeled using their electrical analog. **(B)** unloaded aorta geometry with ***e***_*k*_ (k = 1–4) showing the directions of the four collagen fiber families, **(C)** unloaded LV geometry with fiber directions varying from 60° at the endocardium to −60° at the epicardium wall (all dimensions are in cm).

Finite element models of the aorta and LV were coupled via a closed-loop modeling framework describing the systemic circulatory system. Other components of the circulatory system were modeled using electrical analogs (Figure 1A). Mass of blood was conserved by the following equations relating the rate of volume change in each storage compartment of the circulatory system to the inflow and outflow rates

(1a) $$\frac{dV_{LA}(t)}{dt}=q_{ven}\left(t\right)-q_{mv}\left(t\right);$$

(1b) $$\frac{dV_{LV}(t)}{dt}=q_{mv}\left(t\right)-q_{ao}\left(t\right);$$

(1c) $$\frac{dV_{art}(t)}{dt}=q_{ao}\left(t\right)-q_{per}\left(t\right);$$

(1d) $$\frac{dV_{ven}(t)}{dt}=q_{per}(t)-q_{ven}(t),$$

where *V*_*LA*_, *V*_*LV*_, *V*_*art*_, and *V*_*ven*_ are volumes of each compartment, and *q*_*ven*_, *q*_*mv*_, *q*_*ao*_, and *q*_*per*_ are flow rates at different segments (Figure 1A). Flowrate at different segments of the circulatory model depends on their resistance to flow (*R*_*ao*_, *R*_*per*_, *R*_*ven*_, and *R*_*mv*_) and the pressure difference between the connecting storage compartments (i.e., pressure gradient). The flow rates are given by

(2a) $$q_{ao}(t)=\{\begin{array}{cc} \frac{P_{LV,cav}(t)-P_{art,cav}(t)}{R_{ao}} & when,P_{LV,cav}(t)\geq P_{art,cav}(t)\\ 0 & when,\;P_{LV,cav}(t)<P_{art,cav}(t) \end{array};$$

(2b) $$q_{per}\left(t\right)=\frac{P_{art,cav}\left(t\right)-P_{ven}\left(t\right)}{R_{per}};$$

(2c) $$\;q_{ven}\left(t\right)=\frac{P_{ven}\left(t\right)-P_{LA}\left(t\right)}{R_{ven}};$$

(2d) $$q_{mv}(t)=\left\{ \begin{array}{l} \frac{P_{LA}(t)-P_{LV,cav}(t)}{R_{mv}}when,\;P_{LA}(t)\geq P_{LV,cav}(t)\;\\ \;0\;when,\;P_{LA}(t)<P_{LV,cav}(t) \end{array}\right.$$

Pressure in each storage compartment is a function of its volume. A simplified pressure volume relationship,

(3) $$P_{ven}(t)=\frac{V_{ven}(t)-V_{ven,0}}{C_{ven}},$$

was prescribed for the veins, where *V*_*ven*, 0_ is a constant resting volume of the veins and *C*_*ven*_ is the total compliance of the venous system. On the other hand, pressure in the left atrium *P*_*LA*_(*t*) was prescribed to be a function of its volume *V*_*LA*_(*t*) by the following equations that describe its contraction using a time-varying elastance function *e*(*t*):

(4) $$P_{LA}(t)\;=\;e(t)P_{es,LA}(V_{LA}(t))\;+\;(1-e(t))\;P_{ed,LA}(V_{LA}(t)),$$

where

(5a) $$P_{es,LA}(V_{LA}(t))\;=\;E_{es,LA}(V_{LA}(t)-V_{0,LA}),$$

(5b) $$P_{ed,LA}\left(V_{LA}(t)\right)=A_{LA}\;\left(e^{B_{LA}\left(V_{LA}(t)\;-V_{0,LA}\right)}-1\right),$$

and,

(5c) $$e\left(t\right)=\{\begin{array}{c} \frac{1}{2}\left(\sin\left[\left(\frac{\pi }{t_{max}}\right)t\text{​}-\text{​}\frac{\pi }{2}\right]+\text{​}1\right);\;0<\;t\;\leq \;3/2\;t_{max}\;\\ \frac{1}{2}\;e^{-(t-3/2t_{max})/\tau };\;t>3/2\;t_{max} \end{array}.$$

In Equations (5a,b), *E*_*es, LA*_ is the end-systolic elastance of the left atrium, *V*_0, *LA*_ is the volume axis intercept of the end-systolic pressure volume relationship (ESPVR), and both *A*_*LA*_ and *B*_*LA*_ are parameters of the end-diastolic pressure volume relationship (EDPVR) of the left atrium. The driving function *e*(*t*) is given in Equation (5c) in which *t*_*max*_ is the point of maximal chamber elastance and τ is the time constant of relaxation. The values of *E*_*es, LA*_, *V*_0, *LA*_, *A*_*LA*_, *B*_*LA*_, *t*_*max*_, and τ are listed in Table 1.

**Table 1:** Parameters of time varying elastance model for the left atrium.

**Parameter**	**Unit**	**Values**
End-systolic elastance, *E*_*es, LA*_	Pa/ml	60
Volume axis intercept, *V*_0, *LA*_	ml	10
Scaling factor for EDPVR, *A*_*LA*_	Pa	58.67
Exponent for EDPVR, *B*_*LA*_	ml^−1^	0.049
Time to end-systole, *t*_*max*_	ms	200
Time constant of relaxation, τ	ms	35

Finally, pressure in the other two storage compartments, namely, LV and aorta, depends on their corresponding volume through non-closed form functions

(6) $$P_{LV,cav}(t)\;=\;f^{LV}(V_{LV}(t)),$$

(7) $$P_{art,\;cav}\left(t\right)\;=f^{art}\left(V_{art}\left(t\right)\right).$$

The functional relationships between pressure and volume in the LV and aorta were obtained using the FE method as described in the next section.

### Finite element formulation of the left ventricle and aorta

Finite element formulation of the other two storage compartments can be generalized from the minimization of the following Lagrangian functional with the subscript *k* = *LV* denoting the LV and *k* = *art* denoting the aorta

(8) $$\begin{array}{l} L_{k}\left(uk,p_{k},P_{k,cav},c_{1,k},c_{2,k}\right)=\int_{\Omega_{0,k}}^{}W_{k}\left(uk\right)dV\\ \;-\int_{\Omega_{0,k}}^{}p_{k}\left(J_{k}-1\right)dV-P_{k,cav}\left(V_{k,cav}\left(uk\right)-V_{k}\right)\\ \;-c_{1,k}\cdot \int_{\Omega_{0,k}}^{}uk\;dV-c_{2,k}\cdot \int_{\Omega_{0,k}}^{}Xk\times uk\;dV. \end{array}$$

In the above equation, ***u***_***k***_ is the displacement field, *P*_*k, cav*_ is the Lagrange multiplier to constrain the cavity volume *V*_*k, cav*_(***u***_***k***_) to a prescribed value *V*_*k*_ (Pezzuto and Ambrosi, 2014), *p*_*k*_ is a Lagrange multiplier to enforce incompressibility of the tissue (i.e., Jacobian of the deformation gradient tensor *J*_*k*_ = 1), and both ***c***_**1**, ***k***_ and ***c***_**2**, ***k***_ are Lagrange multipliers to constrain rigid body translation (i.e., zero mean translation) and rotation (i.e., zero mean rotation) (Pezzuto et al., 2014). The functional relationship between the cavity volumes of the LV and aorta to their respective displacement fields is given by

(9) $$V_{k,cav}\left(uk\right)=\;\int_{\Omega_{inner}}^{}dv=-\frac{1}{3}\int_{\Gamma_{inner}}^{}x_{k}.n_{k}\;da\;,$$

where Ω_*inner*_ is the volume enclosed by the inner surface Γ_*inner*_ and the basal surface at *z* = 0, and ***n***_***k***_ is the outward unit normal vector.

Pressure-volume relationships of the LV and aorta required in the lumped parameter circulatory model [i.e., Equations (6, 7)] were defined by the solution obtained from minimizing the functional. Taking the first variation of the functional in Equation (8) leads to the following expression:

(10) $$\begin{array}{c} \delta L_{k}\left(uk,p_{k},P_{k,cav},c_{1,k},c_{2,k}\right)=\int_{\Omega_{0,k}}^{}(Pk\;-\;p_{k}Fk^{-T}):\nabla \delta uk\;dV\\ -\int_{\Omega_{0,k}}^{}\delta p_{k}\left(J_{k}-1\right)dV-P_{k,cav}\int_{\Omega_{0,k}}^{}cof\left(Fk\right):\nabla \delta uk\;dV\;\;\\ -\delta P_{k,cav}\left(V_{k,cav}\left(u_{}k\right)-V_{k}\right)-\delta c1,k\cdot \int_{\Omega_{0,k}}^{}uk\;dV\;\\ -\delta c_{2,k}\cdot \int_{\Omega_{0,k}}^{}Xk\times uk\;dV-c_{1,k}\cdot \int_{\Omega_{0,k}}^{}\delta uk\;dV\;\\ -c_{2,k}\cdot \int_{\Omega_{0,k}}^{}Xk\times \delta u_{}k\;dV. \end{array}$$

In Equation (10), ***P***_***k***_ is the first Piola Kirchhoff stress tensor, ***F***_***k***_ is the deformation gradient tensor, δ***u***_***k***_, δ*p*_*k*_, δ*P*_*k, cav*_, **δ*c***_**1, *k***_, δ***c***_**2**, ***k***_ are the variation of the displacement field, Lagrange multipliers for enforcing incompressibility and volume constraint, zero mean translation and rotation, respectively. The Euler-Lagrange problem then becomes finding $u_{k}\;{\in \;H}^{1}\left(\Omega_{0,k}\right),\;p_{k}\;\in \;L^{2}\left(\Omega_{0,k}\right),P_{k,cav}\;\in \;\mathbb{R},c_{1,k}\;\in \;{\mathbb{R}}^{3},c_{2,k}\in \;{\mathbb{R}}^{3}\;$that satisfies

(11) $$\delta L_{k}\left(uk,p_{k},P_{k,cav},c_{1,k},c_{2,k}\right)=0$$

and ***u***_*k*_.***n***_*k*_|_***base***_ **=** 0 (for constraining the basal deformation to be in-plane) ∀ δ***u***_***k***_ ${\in \;H}^{1}\left(\Omega_{0,k}\right),\;\delta p_{k}\in L^{2}\left(\Omega_{0,k}\right),$ δ$P_{k,cav}\;\in \;\mathbb{R},\;{\delta c}_{1,k}\;\in \;{\mathbb{R}}^{3},\;\delta c_{2,k}\;\in \;{\mathbb{R}}^{3}.$

An explicit time integration scheme was used to solve the ODEs in Equation (1). Specifically, compartment volumes (*V*_*LA*_, *V*_*LV*_, *V*_*art*_, *V*_*ven*_) at each timestep *t*_*i*_ was determined from their respective values and the segmental flow rates (*q*_*ven*_, *q*_*mv*_, *q*_*ao*_, *q*_*per*_) at previous timestep *t*_*i*−1_ in Equation (1). The computed compartment volumes at *t*_*i*_ were used to update the corresponding pressures (*P*_*LA*_, *P*_*LV*_, *P*_*art*_, *P*_*ven*_). Pressures in the left atrium (*P*_*LA*_) and veins (*P*_*ven*_) were computed from Equations (4) and (3), respectively. On the other hand, pressures in the LV (*P*_*LV,cav*_) and aorta (*P*_*art, cav*_) were computed from the FE solutions of Equation (11) (for *k* = *LV* and *art*) with the volumes (*V*_*LV*_, *V*_*art*_) at timestep *t*_*i*_ as input. We note here that (*P*_*LV,cav*_, *P*_*art, cav*_) are scalar Lagrange multipliers in the FE formulation for constraining the cavity volumes to the prescribed values (*V*_*LV*_, *V*_*art*_). The computed pressures at timestep *t*_*i*_ were then used to update the segmental flow rates in Equation (2) that will be used to compute the compartment volumes at timestep *t*_*i*+1_ in the next iteration. Steady-state pressure-volume loop was established by running the simulation over several cardiac cycles, each with a cycle time of 800 ms (equivalent to 75 bpm). All the parameter values used in the circulatory model are listed in Table 2.

**Table 2:** Parameters of the closed loop lumped parameter circulatory framework.

**Parameter**	**Unit**	**Values**
Aortic valve resistance, *R*_*ao*_	Pa ms ml^−1^	2,000
Peripheral resistance, *R*_*per*_	Pa ms ml^−1^	125,000
Venous resistance, *R*_*ven*_	Pa ms ml^−1^	2,000
Mitral valve resistance, *R*_*mv*_	Pa ms ml^−1^	2,000
Venous compliance, *C*_*ven*_	ml Pa	0.3
Resting volume for vein, *V*_*ven*, 0_	ml	3,200

### Geometry and microstructure of the LV

The LV geometry was described using a half prolate ellipsoid that was discretized with 1325 quadratic tetrahedral elements. The helix angle associated with the myofiber direction **e**_**_*f*__0_**_ was varied with a linear transmural variation from 60° at the endocardium to −60° at the epicardium in the LV wall based on previous experimental measurements (Streeter et al., 1969) (Figure 1C).

### Constitutive law of the LV

An active stress formulation was used to describe the LV's mechanical behavior in the cardiac cycle. In this formulation, the stress tensor ***P***_***LV***_ can be decomposed additively into a passive component ***P***_***LV,p***_ and an active component ***P***_***LV,a***_ (i.e., ***P***_***LV***_**=*P***_***LV,a***_**+*P***_***LV,p***_). The passive stress tensor was defined by ***P***_***LV,p***_**=***dW*_*LV*_/*d****F***_***LV***_, where *W*_*LV*_ is a strain energy function of a Fung-type transversely-isotropic hyperelastic material (Guccione et al., 1991) given by

(12a) $$W_{LV}\;=\;\frac{1}{2}C\left(e^{Q}-1\right),$$

where,

(12b) $$\begin{array}{c} Q=b_{ff}E_{ff}^{2}+b_{xx}\left(E_{ss}^{2}+E_{nn}^{2}+E_{sn}^{2}+E_{ns}^{2}\right)\\ +\;b_{fx}\left(E_{fn}^{2}+E_{nf}^{2}+E_{fs}^{2}+E_{sf}^{2}\right). \end{array}$$

In Equation (12), *E*_*ij*_ with (*i, j*) ϵ (*f, s, n*) are components of the Green-Lagrange strain tensor **E**_***LV***_ with *f*, *s, n* denoting the myocardial fiber, sheet and sheet normal directions, respectively. Material parameters of the passive constitutive model are denoted by *C, b*_*ff*_, *b*_*xx*_, and *b*_*fx*_.

The active stress **P**_***LV,a***_ was calculated along the local fiber direction using a previously developed active contraction model (Guccione et al., 1993; Dang et al., 2005),

(13) $$P_{LV},a=T_{max}\frac{Ca_{0}^{2}}{Ca_{0}^{2}+ECa_{50}^{2}}C_{t}\;e_{f}⊗e_{f}{}_{0}.$$

In the above equation, **e**_***f***_ and **e**_**_*f*__0_**_ are, respectively, the local vectors defining the muscle fiber direction in the current and reference configurations, *T*_*max*_ is the isometric tension achieved at the longest sarcomere length and *Ca*_0_ denotes the peak intracellular calcium concentration. The length dependent calcium sensitivity *ECa*_50_ and the variable *C*_*t*_ are given by

(14a) $$ECa_{50}=\frac{{\left(Ca_{0}\right)}_{max}}{\sqrt{\exp\left(B\left(l-\;l_{0}\right)\right)-1}},$$

(14b) $$\;C_{t}=\frac{1}{2}\left(1-\cos\omega \right).$$

In Equation (14a), *B* is a constant, (*C*_*a*_0_)*max*_ is the maximum peak intracellular calcium concentration and *l*_0_ is the sarcomere length at which no active tension develops. The variable ω in Equation (14b) is given by

(15) $$\omega =\{\begin{array}{c} \;\pi \frac{t}{t_{0}},\;0\leq t<t_{0};\\ \pi \frac{t-t_{0}+t_{r}}{t_{r}},\;t_{0}\leq t<t_{0}+t_{r};\\ \;0\;,\;t_{0}+t_{r}\leq t\;. \end{array}$$

In the above equation, *t*_0_ is the time taken to reach peak tension and *t*_*r*_ is the duration of relaxation that depends linearly on the sarcomere length *l* by

(16) $$t_{r}=ml+b,$$

where *m* and *b* are constants. The sarcomere length *l* can be calculated from the myofiber stretch λ_*LV*_ by

(17a) $$\lambda_{LV}=\sqrt{e_{f_{0}}\cdot C_{LV}e_{f_{0}}}\;,$$

(17b) $$\;l=\;\lambda_{LV}l_{r}.$$

In Equation (17a), $C_{LV}={F_{LV}}^{T}F_{LV}$ is the right Cauchy-Green deformation tensor and *l*_*r*_ is the relaxed sarcomere length. Parameter values associated with the LV model are tabulated in Table 3.

**Table 3:** Parameters of the LV model.

**Parameter**	**Description**	**Value**
*C*	material parameter, kPa	0.10
*b_*ff*_*	material parameter	29.9
*b_*xx*_*	material parameter	13.3
*b_*fx*_*	material parameter	26.6
*T_*max*_*	isometric tension under maximal activation, kPa	200.7
*Ca_0_*	peak intracellular calcium concentration, μM	4.35
*(Ca_0_)_*max*_*	maximum peak intracellular calcium concentration, μM	4.35
*B*	governs shape of peak isometric tension-sarcomere length relation, μm^−1^	4.75
*l_0_*	sarcomere length at which no active tension develops, μm	1.58
*t_0_*	time to peak tension, ms	171
*m*	slope of linear relaxation duration-sarcomere length relation, ms μm^−1^	1,049
*b*	time-intercept of linear relaxation duration-sarcomere length relation, ms	1,500
*l*_*r*_	relaxed sarcomere length, μm	1.85

### Geometry and microstructure of the aorta

An idealized geometry of the aorta extending from the heart to the thoracic region from a previous study (Vasava et al., 2012) was used here. The geometry was discretized using 1020 quadratic tetrahedral elements. The aorta diameter was assumed to be constant in the first segment starting from the aortic root to the middle of the aortic arch, and then gradually decreased toward the thoracic region. Aortic wall thickness was kept constant (Figure 1B).

### Constitutive law of the aorta

Stress tensor in the aortic wall was defined by ***P***_***art***_**=***dW*_*art*_/*d****F***_**art**_, where *W*_*art*_ is the sum of the strain energy functions associated with those from the key tissue constituents, namely, elastin-dominated matrix *W*_*e*_, collagen fiber families *W*_*c, k*_ and vascular smooth muscle cells (SMC) *W*_*m*_ (Baek et al., 2007; Zeinali-Davarani et al., 2011), i.e.,

(18) $$W_{art}={\text{W}}_{e}+\sum_{k=1}^{4}W_{c,k}+{\text{W}}_{m}.$$

Strain energy function of the elastin-dominated amorphous matrix is given by

(19) $${\text{W}}_{e}=M_{e}\left(\frac{c_{1}}{2}\right)\left(tr\left(\;C_{art}\right)-3\right),$$

where *M*_*e*_ is the mass per unit volume of the elastin in the tissue, *c*_1_ is a material parameter and, $C_{art}=F_{art}^{T}F_{art}$ is the right Cauchy-Green deformation tensor associated with the aorta.

Four collagen fiber families were considered here. The first and second families of collagen fibers (*k* = 1 and 2) were oriented in the longitudinal and circumferential directions, whereas the third and fourth families of collagen fibers (*k* = 3 and 4) were oriented, respectively, at an angle α = 45° and −45° with respect to the longitudinal axis (Figure 1B). We assumed the same strain energy function for all the families of collagen fibers that is given by

(20) $${\text{W}}_{c,k}\;=M_{k}\frac{c_{2}}{4c_{3}}\left\{\exp\left[c_{3}{\left(\lambda_{k}{}^{2}-1\right)}^{2}\right]-1\right\}.$$

In Equation (20), *M*_*k*_ is the mass per unit volume of *k*th family of collagen fibers, λ_*k*_ is the corresponding stretch of those fibers, and both *c*_2_ and *c*_3_ are the material parameters. The stretch in the *k*th family of collagen fibers was defined by $\lambda_{k}=\sqrt{e_{k0}\cdot C_{art}e_{k0}},$ where **e**_***k*0**_ is the local unit vector defining the corresponding fibers orientation.

Strain energy function of the smooth muscle cells W_*m*_ was additively decomposed into one describing its passive mechanical behavior W_*m, p*_ and one describing its active behavior W_*m, a*_(i.e., W_*m*_ = W_*m, p*_+W_*m, a*_). The passive strain energy function is given by

(21) $${\text{W}}_{m,p\;}=M_{m}\frac{c_{4}}{4c_{5}}\left\{exp\left[c_{5}{\left(\lambda_{m}{}^{2}-1\right)}^{2}\right]-1\right\}.$$

Here, *M*_*m*_ is the mass per unit volume of the smooth muscle in the tissue, λ_*m*_ is the stretch of the smooth muscle, whereas *c*_4_ and *c*_5_ are the material parameters. The smooth muscle cells were assumed to be perfectly aligned in the circumferential direction. Its stretch is therefore equivalent to that of the second family of collagen fibers, i.e., λ_*m*_ = λ_2_. We used the following strain energy function (Zeinali-Davarani et al., 2011) to describe the active tone of vascular smooth muscle,

(22) $${\text{W}}_{m,a\;}=M_{m}\frac{S_{m}}{\rho }\left[\lambda_{m}+\frac{{\left(\lambda_{M}-\lambda_{m}\right)}^{3}}{3{\left(\lambda_{M}-\lambda_{0}\right)}^{2}}\right].$$

In Equation (22), *S*_*m*_ is the stress at maximum contraction, ρ is the density of the tissue, λ_*M*_ is the prescribed stretch at which the contraction is maximum and λ_0_ is the prescribed stretch at which active force generation ceases. Mass per unit volume for the different constituents were calculated using following relations

(23a) $$M_{e}=\phi_{e}\rho ,$$

(23b) $$M_{m}=\phi_{m}\rho ,$$

(23c) $$M_{k}=\phi_{k}\left(1-\phi_{e}-\phi_{m}\right)\rho ,$$

where ϕ_*e*_, ϕ_*m*_, and ϕ_*k*_ denote the mass fraction for elastin, smooth muscle cells and *k*th family of collagen fibers. It was assumed that 20% of the total collagen mass was distributed equally toward the longitudinal and circumferential fiber families and the remaining 80% was distributed equally to the α = 45° and −45° fiber families. Constitutive parameters, mass fraction of each constituents and other parameters of the aorta model are listed in Table 4.

**Table 4:** Parameters of the aorta model.

Elastin	*c*_1_ = 160 kPa, ϕ_*e*_ = 0.306
Collagen families	*c*_2_ = 0.08 kPa, *c*_3_ = 2.54, ϕ_*c*_ = 0.544 (ϕ_1_ = 0.1ϕ_*c*_, ϕ_2_ = 0.1ϕ_*c*_, ϕ_3_ = 0.4ϕ_*c*_, ϕ_4_ = 0.4ϕ_*c*_)
SMC	*c*_4_ = 0.01 kPa, *c*_5_ = 7.28, ϕ_*m*_ = 0.15
Others	ρ = 1050 kg/m^3^, *S*_*m*_ = 54 kPa, λ_*M*_ = 1.4, λ_0_ = 0.8

The coupled LV-aorta modeling framework, including the solving of FE equations associated with the LV and aorta models, was implemented using the open-source FE library FEniCS (Alnæs et al., 2015).

## Results

**Figure 2:**
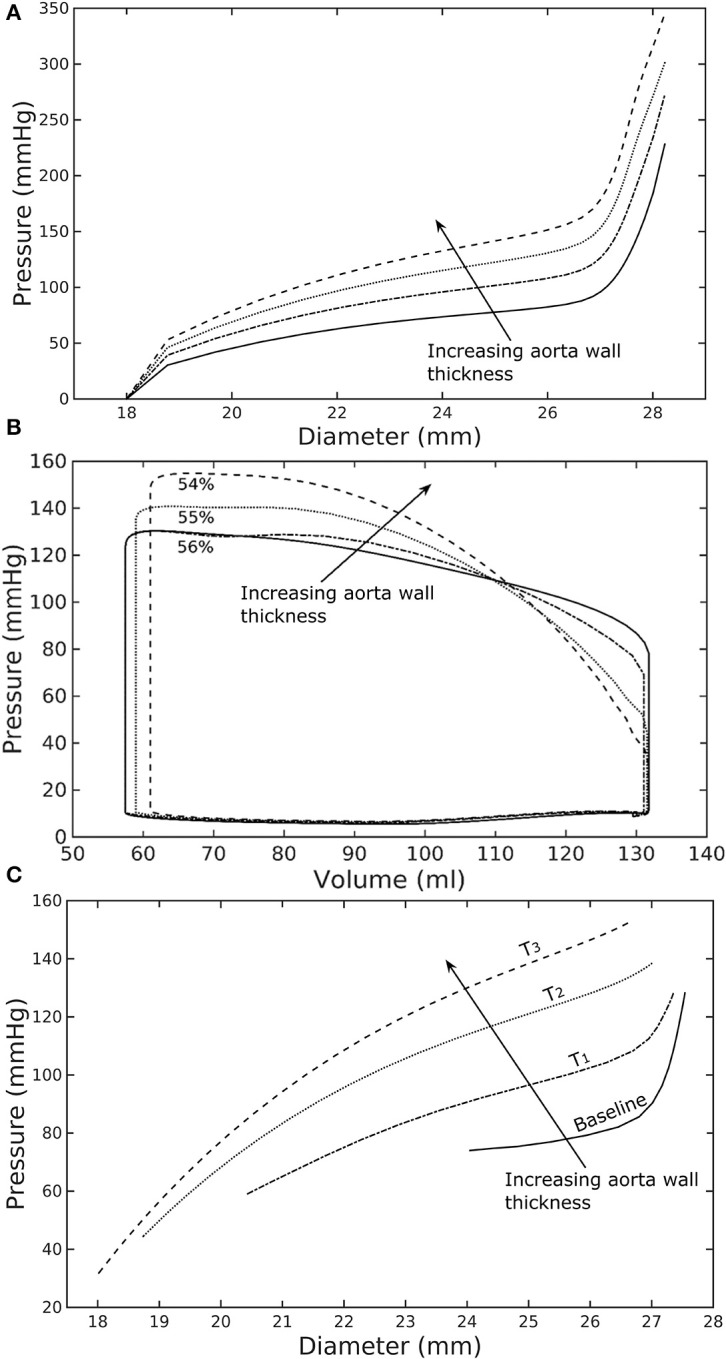
Effects of a change in aorta wall thickness on **(A)** its *ex-vivo* pressure–diameter relationship, **(B)** LV pressure–volume loop and **(C)** pressure—diameter both operating *in-vivo*.

**Figure 3:**
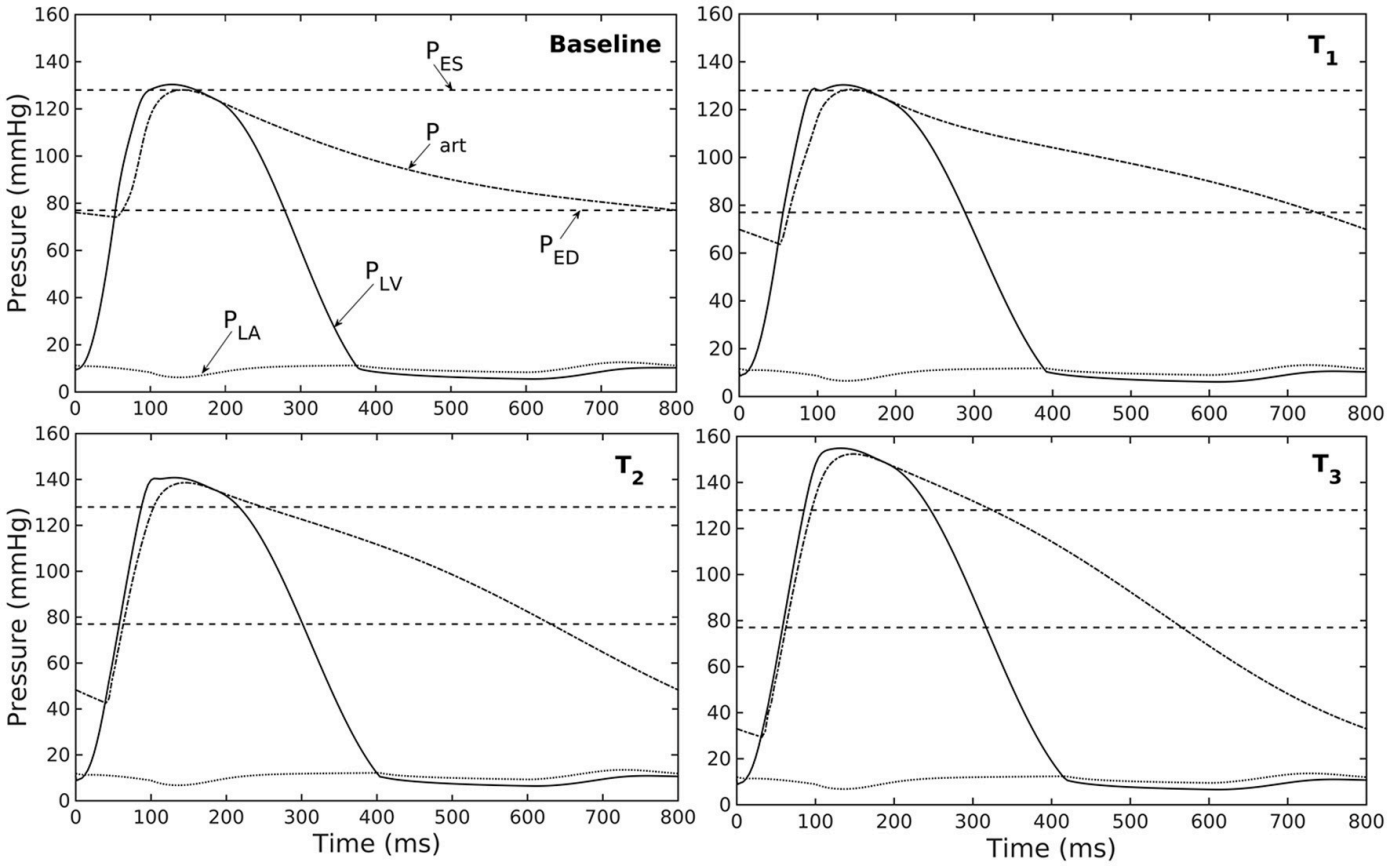
Pressure in LV, aorta, and LA during cardiac cycle with increasing aorta wall thickness in ascending order from the Baseline to T_3_ case (P_ES_ and P_ED_ are respectively, the end-systolic and end-diastolic pressure for the baseline case).

A baseline case was established using the LV-aorta coupling framework so that LV pressure-volume loop and aorta pressure-diameter curve were consistent with measurements in the normal human systemic circulation under physiological conditions. Specifically, model prediction of the LV ejection fraction (EF) was 56%, which is within the normal range in humans (Figure 2B). Similarly, end-diastolic (ED) and end-systolic (ES) diameters of the aorta in the baseline case (Figure 2C) were comparable to *in-vivo* measurements (Greenfield and Patel, 1962; Muraru et al., 2014). We note here that diameter of the aorta mentioned in subsequent text refers to its inner diameter. Pressure waveforms of the LV, aorta, and LA (Figure 3) in the baseline case were also within the normal range with an aortic pulse pressure of 50 mmHg (systolic: 128 mmHg, diastolic: 78 mmHg).

### Effects of a change in aorta wall thickness

Varying the wall thickness in the aorta model led to changes in not only the aorta mechanical behavior but also the LV function (Figure 2). The aorta became stiffer (less compliant) with increasing wall thickness as reflected by an increase in the slope of the pressure-diameter curves (Figure 2A). When operating *in vivo* as simulated in the LV-aorta coupling framework, increasing the aorta wall thickness led to a lower LV EF, a higher peak systolic pressure of the LV (Figure 2B) and a leftward shift in the aorta pressure—diameter relationship with smaller diameter at ED and ES (Figure 2C). Specifically, an increase in aorta ED wall thickness from 1.8 mm (baseline) to 5.4 mm (T_3_ case) was accompanied by an increase in pulse pressure from 50 mmHg (in the baseline case) to 120 mmHg. In comparison, the mean aortic pressure changed by only about 10 mmHg (decreased from 102 to 93 mm Hg) for the same increase in wall thickness.

### Effect of changes in mass fractions of the aorta constituents

**Figure 4:**
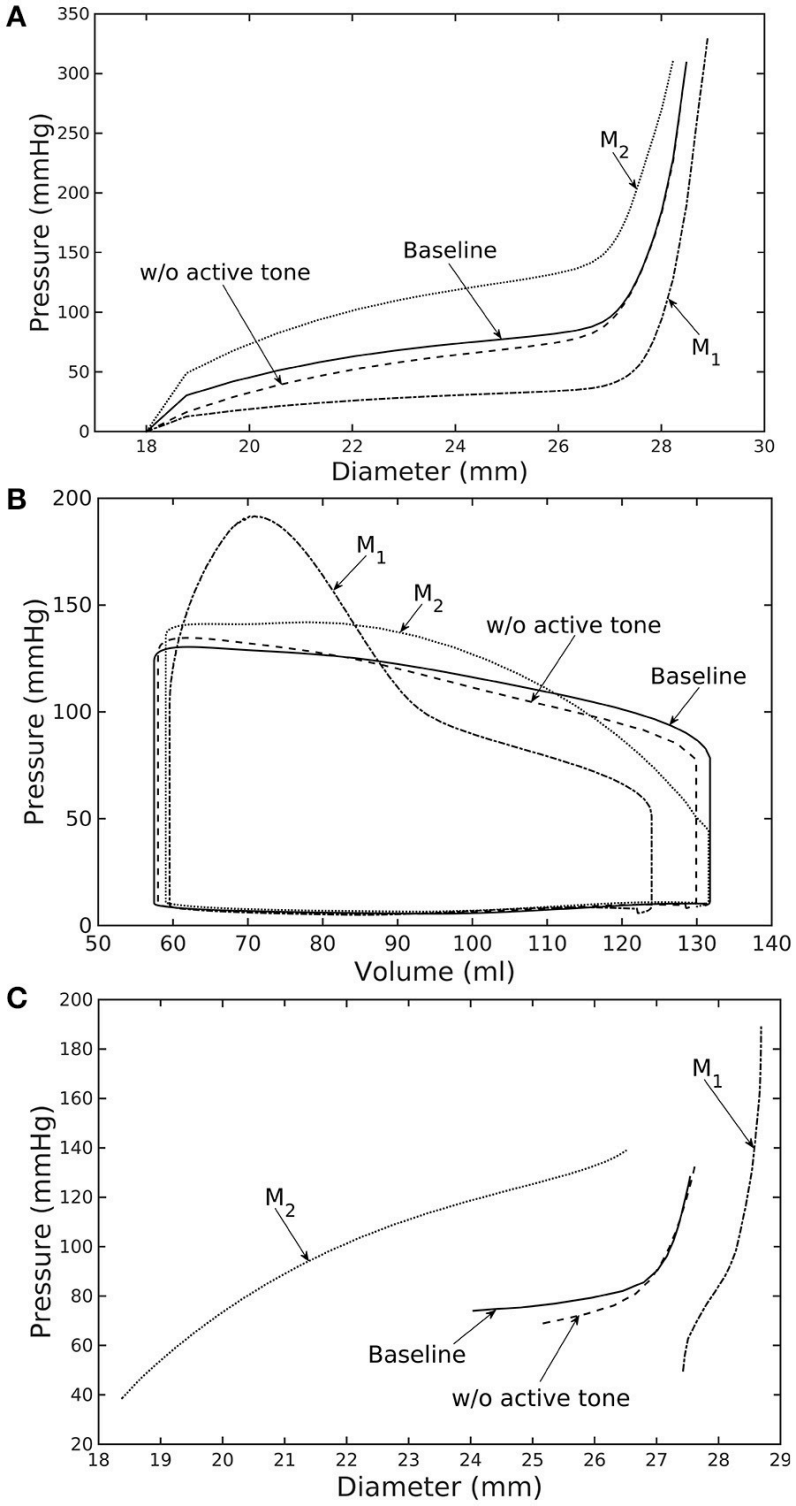
Effects of active tone and aorta constituent mass fractions on **(A)** its *ex-vivo* pressure–diameter relationship, **(B)** LV pressure–volume loop and **(C)** pressure—diameter both operating *in-vivo*. (Refer to Table 5 for cases).

**Figure 5:**
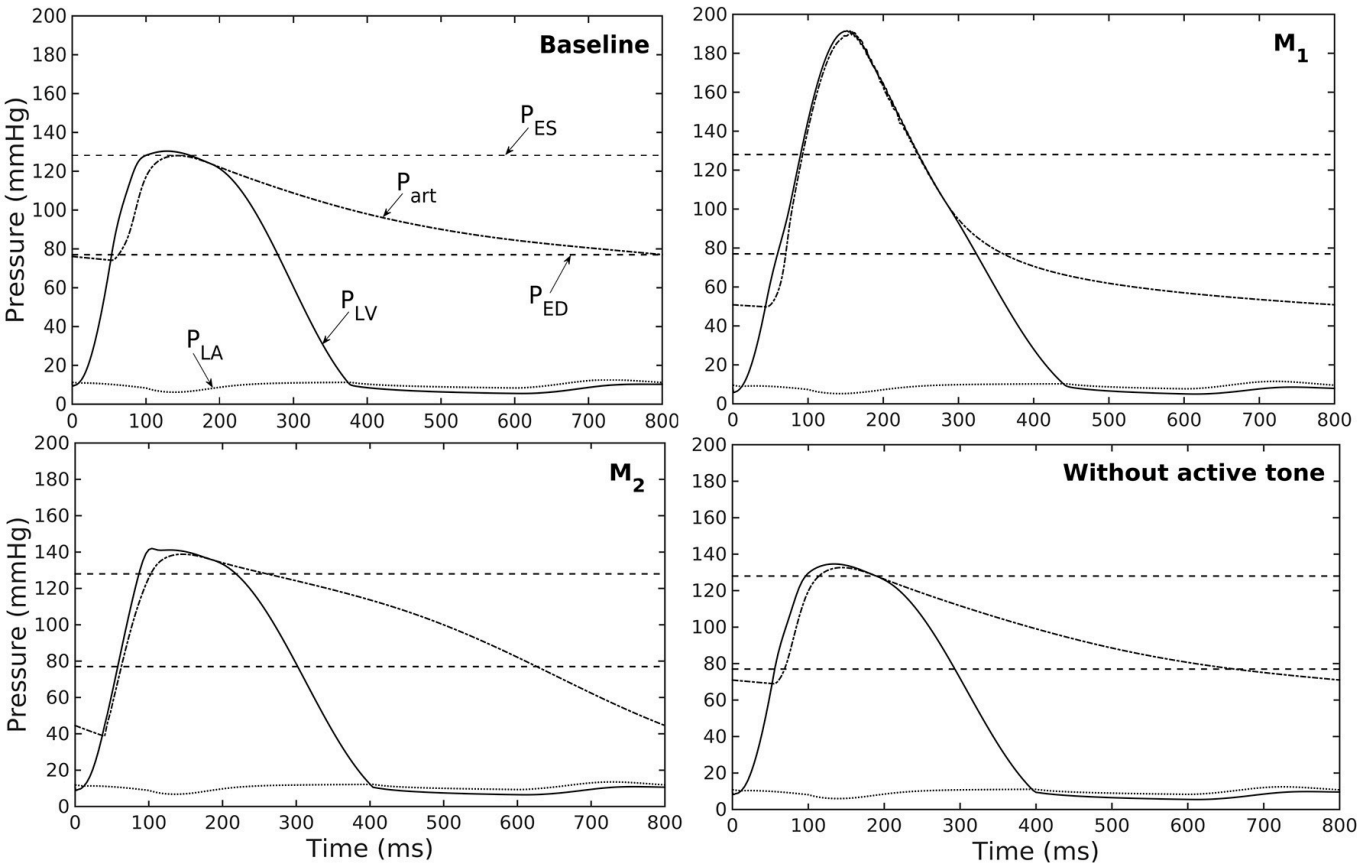
Pressure in LV, aorta, and LA during cardiac cycle for different aorta constituent mass fractions and active tone (P_ES_ and P_ED_ are respectively, the end-systolic and end-diastolic pressure for the baseline case).

Similarly, varying mass fraction of the constituents in the aorta wall (see Table 5 for the different cases) also led to changes in both the aorta and LV functions. Increasing collagen mass fraction with a corresponding decrease in SMC and elastin mass fractions (case M_1_) led to a predominantly exponential pressure - diameter response of the aorta that became extremely steep at larger diameter (i.e., >28 mm) (Figure 4A). This is because the collagen fibers are stiffer than other constituents at large strain. Under *in vivo* operating condition (as simulated in the LV-aorta coupling framework), an increase in collagen mass fraction resulted in a higher peak systolic pressure and a reduced LV EF (Figure 4B). The exponential mechanical response (shown in Figure 4A) of the aorta with higher collagen mass fraction was also reflected in the ejection phase of the LV pressure-volume loop, where the pressure-volume curve became steeper toward end-of-systole. With a higher collagen mass fraction, the aorta also operated at a larger diameter than the baseline *in vivo* (Figure 4C). Pulse pressure in the aorta with higher collagen mass fraction was much higher and decayed more rapidly when compared to the baseline case (Figure 5).

**Table 5:** Mass fractions of the aorta constituents for different cases investigated in the study.

**Case**	**Mass fractions of the constituents**	**Comment**
Baseline	Same as Table 4	No change
Without active	Same as Table 4	No active tone in SM, *S*_*m*_ = 0
M_1_	ϕ_*e*_ = 0.122, ϕ_*m*_ = 0.061, ϕ_*c*_ = 0.816	Collagen increased by 50%, Elastin and SMC decreased proportionally
M_2_	ϕ_*e*_ = 0.49, ϕ_*m*_ = 0.24, ϕ_*c*_ = 0.272	Collagen decreased by 50%, Elastin and SMC increased proportionally

*For collagen fibers, the distribution of mass in four collagen fiber families was kept the same, i.e., ϕ_1_ = 0.1ϕ_c_, ϕ_2_ = 0.1ϕ_c_, ϕ_3_ = 0.4ϕ_c_, ϕ_4_ = 0.4ϕ_c_ for all cases*.

Conversely, reducing collagen mass fraction and increasing elastin and SMC mass fraction proportionally (case M_2_) led to a dominant neo-Hookean type pressure - diameter behavior, particularly, at smaller diameter (<25 mm). Under *in vivo* operating condition, the peak pressure increased slightly but EF remained nearly unchanged in the LV (Figure 4B). The aorta also appeared to be more compliant *in vivo* with a larger change in aortic diameter (~8.1 mm), especially when compared to case M_1_ that has a higher collagen mass fraction (~1.3 mm) (Figure 4C). On the other hand, the aorta also operated at smaller ED and ES diameters than the baseline. Pressure waveforms of the aorta, LV, and LA were not significantly changed compared to the baseline (Figure 5).

In the absence of SMC's active tone, the aorta became slightly more compliant than the baseline at diameter smaller than 27 mm (Figure 4A). Thus, for a given pressure, the diameter was larger than the baseline. Under *in vivo* operating condition, this change led to a slight increase in the LV and aorta pressure at ES than the baseline (Figures 4B,C). On the other hand, LV and aorta pressure at ED decreased without the active tone (Figures 4B,C), resulting in an increase in the aortic pulse pressure compared to baseline (Figure 5).

### Effects of a change in LV contractility

**Figure 6:**
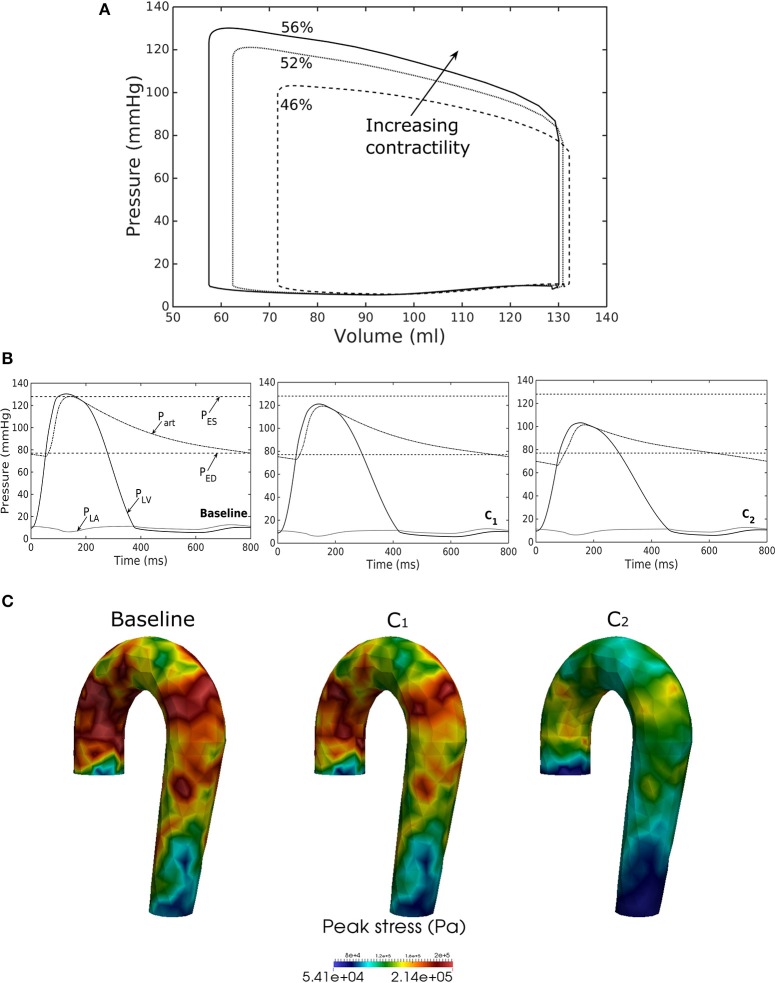
Effects of changes in LV contractility on **(A)** pressure-volume loop, **(B)** pressure waveform in LV, aorta, and LA during cardiac cycle, and **(C)** peak stress in aorta. Contractility decreases in the following order: Baseline, C_1_, C_2_.

Reducing LV contractility (*T*_*max*_) led to a decrease in its peak systolic pressure, end systolic volume and EF (Figure 6A). Pressure also dropped accordingly (Figure 6B) in the aorta together with the peak stress (Figure 6C). With a reduction in LV contractility by 50% (from 200.7 to 100.4 kPa), aorta peak stress was reduced by about 50% compared to the baseline case (from 214 to 110 kPa). The stress was calculated as a root of the sum of the square of all components of the Cauchy stress tensor. Reducing LV contractility also led to changes in the aorta diameter. As a result of lower LV contractility, the aortic pressure decreased that led to less expansion and a decrease in both its ED (from 24.0 mm in baseline to 22.5 mm in case C_2_) and ES diameter (from 27.5 mm in baseline to 27.0 mm in case C_2_).

### Effects of a change in LV passive stiffness

**Figure 7:**
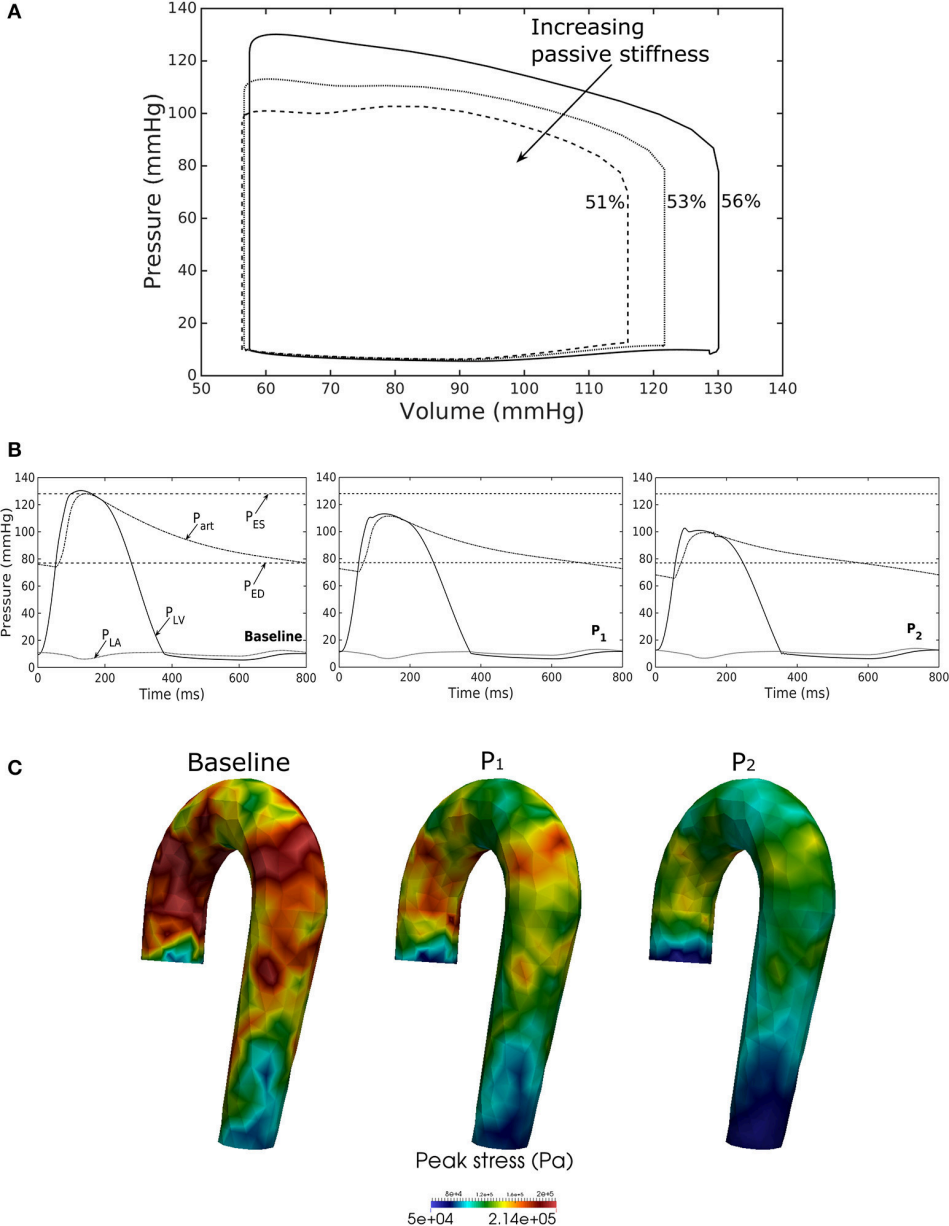
Effects of changes in LV passive stiffness on **(A)** pressure-volume loop, **(B)** pressure waveform in LV, aorta, and LA during cardiac cycle, and **(C)** peak stress in aorta. Passive stiffness increases in the following order: Baseline, P_1_, P_2_.

Increasing the LV passive stiffness (parameter *C*) in Equation (12a) led to a stiffer end diastolic pressure—volume relationship that was accompanied by a reduction in preload, peak systolic pressure, and EF (as end systolic volume remained nearly unchanged) in the chamber (Figure 7A). These changes were translated to a decrease in aortic pressure and peak stress (Figures 7B,C) as well as a reduction in its ED (from 24.0 mm in baseline to 22.4 mm in case P_2_) and ES (from 27.5 mm in baseline to 27.1 mm in case P_2_) diameters.

## Discussion

Finite element models of the LV have been widely used in the literature to study its mechanics as well as organ-scale physiological behaviors in the cardiac cycle (Usyk et al., 2002; Kerckhoffs et al., 2007; Lee et al., 2016; Xi et al., 2016; Shavik et al., 2017). In these models, the aorta is usually represented within the lumped parameter circulatory model by its electrical analog, which cannot separate the effects its geometry, microstructure, and constituents' mechanical behavior have on the LV's operating behavior *in vivo* and vice versa. To the best of our knowledge, this is the first computational modeling framework in which FE models of the aorta and LV are coupled in a closed-loop fashion. This framework enables us to take into detailed account of the geometrical, microstructural, and mechanical behavior of the LV and aorta. We have shown here that the coupled LV—aorta FE framework is able to capture physiological behaviors in both the LV and aorta that are consistent with *in vivo* measurements. We also showed that the framework can reasonably predict the effects of changes in geometry and microstructural details the two compartments have on each other over the cardiac cycle.

Using a detailed FE model of the aorta has enabled us to separate the contributions of the key load bearing constituents (elastin, collagen fibers, and SMCs) have on its mechanical behavior. The aorta FE model predicted a pressure—diameter response in which the mechanical behavior of each constituent is clearly detectable (Figure 2A). For instance, mechanical behavior of aorta at lower diameter range (low stretch) is relatively compliant as it is largely endowed by elastin, but exhibits a very stiff behavior at the higher diameter range (high stretch) when more collagen fibers are recruited. The behavior is consistent with previous experimental studies (Roach and Burton, 1957; Schriefl et al., 2012; Kohn et al., 2015). The pressure—diameter relationship predicted by our model (Figure 2A) resembled a S-shaped curve with a very stiff response after the inflection point that is a typical feature of large proximal arterial vessels (Bader, 1967; Towfiq et al., 1986).

Our model predicted that an increase in aortic wall thickness led it to become more constricted with smaller ED and ES diameter under *in vivo* operating conditions when coupled to the LV in our modeling framework (Figure 2C). Systolic blood pressure and pulse pressure in the aorta increased as a result, and was accompanied by a reduction in stroke volume and an increase in LV peak systolic pressure (Figure 2B). Although previous vascular studies suggest that an increase in arterial wall thickness (that may be accompanied by an increase in stiffness) is a result from an increase blood pressure during aging, more recent evidence have suggested that stiffening is a cause for the increase in blood pressure in which a positive feedback loop between them proceeds gradually (Humphrey et al., 2016). These features are consistent with those found in clinical and experimental studies. Specifically, it has been found that the mean aortic wall thickness increases with age (Li et al., 2004; Rosero et al., 2011) in human, which increases the risk of hypertension and atherosclerosis. Similarly, our model predicted that an increase in aortic wall thickness by 70% elevates the aortic pressure over the hypertensive range (>140 mmHg) (Figure 3).

Changes in the aorta microstructure is a feature of pathological remodeling as well as aging. In the systemic vasculature, the proximal aorta has a compliant behavior that helps to keep the systolic blood pressure down. With aging, however, elastin degenerates and is replaced (i.e., compensated) by collagen in the aorta wall (Schlatmann and Becker, 1977; Tsamis et al., 2013; Kohn et al., 2015). Consequently, the collagen fibers bear more of the load that substantially increases the aorta wall stiffness, especially at high stretch. A stiffer aorta leads to many adverse effects including elevated systolic and pulse pressure during ejection, faster decay in the aortic pressure waveform during diastole, and an increase in ventricular afterload that reduces the LV EF (Borlaug and Kass, 2011). These behaviors are all captured in our framework when collagen and elastin mass fractions were increased and decreased, respectively. Specifically, these microstructural changes led to a reduction in EF (Figure 4B) and an increase in the aortic systolic and pulse pressure with a faster decay of aortic pressure waveform (Figure 5). Our framework also predicted that the aorta underwent more expansion *in vivo* and have a larger operating diameter when collagen mass fraction increases (Figure 4C), which is another key characteristic of aging (Bader, 1967; Mao et al., 2008; Craiem et al., 2012). Interestingly, changes in the aorta collagen mass fraction (that lead to it stiffen at high stretch) also affects the shape of the LV pressure volume loop (Figure 4B, case M_1_). Specifically, a rapid steepening of the LV pressure-volume curve near end-of-systole is predicted by our model when collagen mass fraction is increased. This result suggests that the shape of the LV pressure-volume loop may also reflect, to some extent, the accumulation of collagen fibers in the aorta during remodeling.

Our framework also predicted how changes in the contractility and passive stiffness of the LV affects the aorta function. A decrease in LV contractility led to lower LV EF, lower aortic systolic and pulse pressures, as well as a reduction in the aorta peak stress during the cardiac cycle (Figure 6). A change in contractility (or inotropic state of the myocardium) produced expected changes (Katz, 1988; Burkhoff, 2005) in the LV pressure—volume loop and aortic pulse pressure. Similarly, the model predicted results from a change in the LV passive stiffness that are consistent with experimental observations (Figure 7). With increasing passive stiffness, LV EF decreases and is accompanied by a corresponding decrease in aortic systolic and pulse pressure, as well as peak stress. A change in the passive stiffness of LV (due to such as an alteration in lusitropy), also shows similar changes in the LV pressure—volume loops (Katz, 1988; Burkhoff, 2005) as well as the aortic pressure.

Most clinical studies focus either on the behavior of the LV or aorta. While a number of studies have investigated ventricular—arterial coupling (Kawaguchi et al., 2003; Borlaug and Kass, 2011; Antonini-Canterin et al., 2013; Ky et al., 2013), simplified indices (such as the ratio of end-systolic volume to stroke volume) were used in them to describe this coupling. It is, however, impossible to separate the contribution of microstructure, mechanical behavior, and geometry of the aorta (e.g., diameter or thickness) and LV to any changes in ventricular—arterial coupling. The framework described here helps overcome this limitation and may be useful for developing more insights of the ventricular—arterial interaction. This framework will be extended in future to include the pulmonary vasculature for a more complete understanding of the interactions between the heart and vasculature under different physiological or pathological conditions.

## Model limitations

We have shown that our coupled LV-aorta FE modeling framework is capable of predicting behaviors that are consistent with measurements. There are, however, some limitations associated with our model. First, idealized geometries were used to represent the aorta and LV models. The idealized half-prolate geometry of the LV used here neglected any asymmetrical geometrical features while the aorta geometry was also simplified and had uniform wall thickness. Because wall thickness decreases slightly along the aorta (Mello et al., 2004), its displacement with the given material parameters may be under-estimated. Second, we have assumed homogeneous material properties in our models. Given that studies have suggested that the mechanical properties may be inhomogeneous in the aorta (Kermani et al., 2017) and LV (Khokhlova and Iribe, 2016), the prescribed material parameters are bulk quantities. While thoracic aortic wall thickness and its stiffness varies, previous experimental studies reported that the aortic structural stiffness (product of intrinsic stiffness and aortic wall thickness) is relatively uniform in the circumferential and longitudinal directions (Kim and Baek, 2011; Kim et al., 2013). Third, the dynamical behavior of fluid and its interaction with the vessel wall were neglected here, and as such, the framework did not take into account the spatial variation of pressure waveform along the aortic tree and shear stress on the luminal surface of the vessel. However, we do not expect this limitation to severely affect our result because wall shear stress in the human aorta (~50 dyn/cm^2^ or 0.037 mmHg) (LaDisa et al., 2011) is substantially lower than the pressure (normal stress) (60–120 mm Hg), and the arterial pressure increases by only about 10% from the ascending to the abdominal aorta (Smulyan and Safar, 1997). Fourth, a rule based myofiber orientation in which the helix angle varies linearly across the myocardial wall was used to describe the LV microstructure. Fifth, remodeling of the aorta and LV was simulated by directly manipulating the parameters without consideration of any growth and remodeling mechanisms. Last, we have considered only systemic circulation in this model and ignored the presence of the right ventricle and pulmonary circulatory system that may affect LV and aorta mechanics.

## Author contributions

SMS and LCL developed the theoretical formulation and computational framework of the model. ZJ and SB helped on the development of the theoretical formulation. SMS carried out the simulations for different cases and prepared the results. All authors helped in interpretation of the results and contributed to the final manuscript.

### Conflict of interest statement

The authors declare that the research was conducted in the absence of any commercial or financial relationships that could be construed as a potential conflict of interest.
